# Depressive Symptoms in Crohn's Disease: Relationship with Immune Activation and Tryptophan Availability

**DOI:** 10.1371/journal.pone.0060435

**Published:** 2013-03-27

**Authors:** Sinan Guloksuz, Marieke Wichers, Gunter Kenis, Maurice G. V. M. Russel, Annick Wauters, Robert Verkerk, Baer Arts, Jim van Os

**Affiliations:** 1 Department of Psychiatry and Psychology, Maastricht University Medical Centre, EURON, Maastricht, The Netherlands; 2 Department of Gastroenterology and Hepatology, Medisch Spectrum Twente, Enschede, The Netherlands; 3 Laboratory of Clinical Biology, ZNA Middelheim, Antwerp, Belgium; 4 Laboratory of Medical Biochemistry, University of Antwerp, Wilrijk, Belgium; 5 King's College London, King's Health Partners, Department of Psychosis Studies, Institute of Psychiatry, London, United Kingdom; Catholic University of Sacred Heart of Rome, Italy

## Abstract

Crohn's disease (CD) is associated with immune activation and depressive symptoms. This study determines the impact of anti-tumor necrosis factor (TNF)-α treatment in CD patients on depressive symptoms and the degree to which tryptophan (TRP) availability and immune markers mediate this effect. Fifteen patients with CD, eligible for anti-TNF-α treatment were recruited. Disease activity (Harvey-Bradshaw Index (HBI), Crohn's Disease Activity Index (CDAI)), fatigue (Multidimensional Fatigue Inventory (MFI)), quality of life (Inflammatory Bowel Disease Questionnaire (IBDQ)), symptoms of depression and anxiety (Symptom Checklist (SCL-90), Beck Depression Inventory (BDI), Hamilton Depression Rating Scale (HDRS)), immune activation (acute phase proteins (APP)), zinc and TRP availability were assessed before treatment and after 2, 4 and 8 weeks. Anti-TNF-α increased IBDQ scores and reduced all depression scores; however only SCL-90 depression scores remained decreased after correction for HBI. Positive APPs decreased, while negative APPs increased after treatment. After correction for HBI, both level and percentage of γ fraction were associated with SCL-90 depression scores over time. After correction for HBI, patients with current/past depressive disorder displayed higher levels of positive APPs and lower levels of negative APPs and zinc. TRP availability remained invariant over time and there was no association between SCL-90 depression scores and TRP availability. Inflammatory reactions in CD are more evident in patients with comorbid depression, regardless of disease activity. Anti-TNF-α treatment in CD reduces depressive symptoms, in part independently of disease activity; there was no evidence that this effect was mediated by immune-induced changes in TRP availability.

## Introduction

Crohn's disease (CD) has been associated with an increased prevalence of psychopathology. In addition, it is proposed that CD is more likely to occur in subjects with predisposing personality traits, such as high level of neuroticism and introversion [1]. Previous studies show mixed results concerning the temporal relationship between the onset of gastrointestinal complaints and symptoms of mental disorder [2], [3], [4], [5], [6], [7], [8]. However, once present, bouts of disease activity and symptoms of anxiety and depression tend to co-occur. Elevated levels of inflammatory mediators have been implicated in the pathophysiology of CD. The immune response in CD patients typically has been considered majorly as Th1-type, assessed by elevated expression of interleukin (IL)-12, tumor necrosis factor (TNF)-α and interferon (IFN)-γ, which are pro-inflammatory cytokines that increase macrophage and natural killer cell activation, antigen presenting cell function, and lead to the production of other pro-inflammatory mediators [9], [10], [11]. In addition, the role of Th-17-mediated response in pathophysiology of CD has recently been implicated [11].

Considering that the presence of depression predicts lower remission rates and decreases the time to retreatment of CD [12], it is plausible to hypothesize that there is an interaction between depression and CD. A growing body of evidence also supports the notion that immune-modulation plays a role in pathogenesis of depression [13], [14], [15]. Administration of the pro-inflammatory cytokine IFN-α in humans triggers the development of depressive symptoms in up to 45% of participants [16]. Furthermore, increased production of IL-6, IL-1β, IFN-γ and TNF-α, as well as signs of an acute phase response, i.e. increased production of positive acute phase proteins (APPs) and decreased production of negative APPs, are associated with depression [13], [14], [17], [18], [19], [20], [21]. The observation of higher levels of positive APPs accompanied by low levels of negative APPs supports the notion that depression is inflammatory-related. Likewise, zinc, which plays a role in inflammatory mechanisms as a key antioxidant, has been reported at reduced levels in patients with depression [21], [22]. In addition, increased levels of complement factors C3c and C4, as well as immunoglobulin M (IgM) and IgG are also observed in this disorder [23]. Immune activation may impact on mood, by decreasing the availability of peripheral tryptophan (TRP) that may cross the blood-brain-barrier [13], [14], [15]. TRP is the precursor of serotonin (5-HT), a neurotransmitter synthesized in the brain and important in the regulation of mood. Earlier studies show that indicators of the availability of TRP to brain, serum/plasma TRP, as well as the ratio of TRP to the sum of competing amino acids (CAA)**—** known to compete for the cerebral uptake mechanism of TRP **—**, are lower in depression [21], [24], [25], [26], [27], [28].

The causal mechanism underlying the relationship between CD and mental disorder is unclear. Symptoms of depression and anxiety may represent the psychological response to disease activity. On the other hand, it may be hypothesized that activation of the inflammatory immune response causes both CD and symptoms of mental disorder, which could explain (i) the uncertain temporal relationship between onset of CD and symptoms of mental disorder, (ii) the close relationship between disease state and psychopathology, and (iii) personality differences between CD patients and controls. There is some evidence that depressive symptoms may be reduced in CD patients after infusion of anti-TNF-α, an efficient treatment for gastrointestinal symptoms in CD [12], [29].

Therefore, the aim of the current study was to determine the impact of anti-TNF-α treatment in CD patients on depressive symptoms, and to examine the possibility that improvement of depressive symptoms occurs in parallel with changes in TRP availability imposed by a reduction in inflammation as reflected by levels of APPs.

## Methods

### Subjects

15 patients (4 men and 11 women) with CD, eligible for anti-TNF-α, infliximab (Remicade®) infusion were recruited. Inclusion criteria were: having a Harvey-Bradshaw Index (HBI) score>10 or active perianal fistula, being allergic or not responding to the following treatments: azathioprine, methotrexate and/or corticosteroids. Exclusion criteria were: age <18 and >65 years, pregnancy or intention to get pregnant within the period of treatment and up to 6 months after discontinuation of therapy, women not practicing or not willing to practice safe methods of contraception during the treatment period up to 6 months after discontinuation of therapy, lactation, human immunodeficiency virus positivity, chemotherapy or systemic antiviral treatment during the 6 months prior to study entry, presence of other serious disease (e.g. malignancy, uncontrolled myocardial disease or severe arrhythmias), tuberculosis positivity (current or past), creatinine levels over 150 mmol/L or 1.70 mg/dl, any condition which in the opinion of the (co-) investigator might interfere with the evaluation of the study objectives, patients meeting axis I criteria for mental disorders as defined by DSM-IV, except for patients meeting the criteria for a depressive or anxiety disorder.

The study was approved by the standing Medical Ethics Committee of Maastricht University, and carried out in accordance with the Declaration of Helsinki (Hong Kong Modification, 1989). Written informed consent was obtained from each subject prior to participation.

### Study design

A within-subject design was used to determine (i) the effect of anti-TNF-α infusion on disease activity and markers of immune activation, (ii) effect of anti-TNF-α infusion on mood, fatigue and quality of life, (iii) effect of altered immune activation on TRP levels, and (iv) effect of TRP levels on depressive symptoms.

Patients received an infusion of anti-TNF-α, infliximab (Remicade®) (5 mg/kg bodyweight). Psychological assessments and measurements of immune activation were performed before infusion (baseline) and 2, 4 and 8 weeks after infusion. Fasting blood samples were collected between 8.00 and 10.00 AM. Blood samples were centrifuged at 1300x *g* for 10 minutes. Serum was then stored at −80°C until assayed.

### Disease activity and psychological assessments

Disease activity was assessed using the HBI [30] and the Crohn's Disease Activity Index (CDAI) [31]. The latter was assessed only at baseline, 4 and 8 weeks after infusion. Fatigue was assessed by the Multidimensional Fatigue Inventory (MFI), which is a self-report instrument consisting of five scales measuring general fatigue, physical fatigue, reduced activity, reduced motivation and mental fatigue [32]. In order to limit the number of tests applied, only core fatigue dimensions of physical and mental fatigue were included in the analyses in order to avoid overlap with depression dimensions of reduced activity and motivation. Quality of Life was measured using the Inflammatory Bowel Disease Questionnaire (IBDQ) [33]

The presence of depressive symptoms was assessed by the 17-item Hamilton Depression Rating Scale (HDRS) [34]. In addition, symptoms of depression and anxiety were assessed by self-report using the Beck Depression Inventory (BDI) [35], [36] and the Symptom Checklist (SCL-90) [37]. To determine the presence of axis I mental disorder, the Structured Clinical Interview for DSM-IV axis I Disorders Version 5.0 was administered.

### Determination of markers of immune activation and TRP/CAA

Immune activation was determined by using positive APPs (α_1_–antitrypsin, α_1_–acid glycoprotein and α_1_–antichymotrypsin, haptoglobin, ceruloplasmin, fibrinogen, complement factor C3), negative APPs (albumin, transferrin) and zinc as markers of inflammation described in more detail below. Five major fractions of serum proteins were determined using the capillary zone electrophoresis technique; albumin (negative APP), α_1_, α_2_, β and γ globulin fractions. α_1_–antitrypsin, α_1_–acid glycoprotein and α_1_–antichymotrypsin, all positive APPs, migrate in the α_1_ zone, while haptoglobin and ceruloplasmin, also positive APPs, migrate in the α_2_ zone. Fibrinogen, complement factor C3 and transferrin migrate in the β zone and immunoglobulins in the γ zone. Total serum protein determination and electrophoresis for measurement of albumin and the α, β and γ fractions were performed as described previously [38]. Transferrin concentration was analysed using immunoturbidimetry. Serum zinc levels were determined using atomic absorption spectroscopy [22]. Total TRP and CAA were assayed using high-performance liquid chromatography as explained previously [39].

### Statistical analyses

Normality of distribution was ascertained by Kolmogorov-Smirnov test. Levene statistics were used to check the assumption of homogeneity of variances. Distributions of transferrin and percentages of the α1 and α2 fractions were log-transformed to improve normality. The data were analyzed with the Stata computer program, version 12 (Stata Corporation, College Station, TX, USA). Multilevel random regression models were fitted testing main effects and interactions, using the XTREG routine in Stata. The XTREG procedure takes into account the fact that level-one units (repeated observation level) were hierarchically clustered into level-two units (subject level). Effect sizes of explanatory variables were expressed as regression coefficients (B) from the multilevel models. Regression analyses were performed to examine the effects of time (linear and factored) on the CDAI, mental and physical fatigue, dimensions of the IBDQ, HDRS and on the anxiety and depression scales of the SCL-90 questionnaire. Effects of time on immune parameters were similarly estimated. In addition, regression analyses were used to determine associations, over time, between immune parameters and TRP/CAA ratio and between TRP/CAA ratio and measures of depression. Analyses were corrected *a priori* for age, smoking, sex, use of oral contraceptives, medication use during the study (mesalamine, corticosteroids, azathioprine and/or methotrexate), presence of past/current depression or anxiety disorder (0 = not present, 1 = present). In addition, the effect over time of past or current depression or anxiety disorder on immune parameters and TRP/CAA ratio was also investigated. Two-sided statistical significance was set at p<0.05. Each hypothesis was corrected using the Simes' modification of the Bonferroni procedure for multiple testing [40]. Hereafter, the notation for corrected p-values is: p_Simes_.

## Results

### Subject characteristics and missing data

The sample consisted of 15 patients, 4 men and 11 women. The mean age was 32.7 (SD = 11.4) years. Six were cigarette smokers. Seven of 11 women used oral contraceptives. All patients, except one, received concomitant medication. Ten patients were on mesalamine medication, nine used corticosteroids, seven received azathioprine and two received methotrexate medication. Six of 15 patients received the anti-TNF-α infusion for the first time. Two patients fulfilled criteria for lifetime depressive disorder and two for current depressive disorder with melancholic features. Table 1 shows disease activity and assessment of behavioral and psychopathological parameters at each time point. Immune parameters and TRP/CAA at each time point are presented in Table 2. All patients completed the study. With respect to the administered questionnaires, one observation was missing. Six observations were missing in the measurements of immune parameters (10%). Because data were missing randomly, we used the Expectation Maximization (EM) method to impute missing data.

**Table 1:** Total scores of disease activity and psychopathological and behavioural assessments at each time point.

	Baseline	Week 2	Week 4	Week 8
HBI	8.78 (2.91)	3.33 (2.26)	3.73 (2.81)	6.03 (5.68)
CDAI	230.83 (94.75)	NA	102.02 (66.24)	144.84 (105.71)
MFI Physical Fatigue	13.87 (3.64)	11.53 (4.81)	12.20 (5.35)	11.86 (5.36)
MFI Mental Fatigue	9.40 (5.62)	7.20 (3.10)	8.27 (3.94)	9.04 (5.29)
IBDQ Bowel Symptoms	41.87 (6.00)	58.13 (6.28)	58.80 (6.10)	53.18 (12.05)
IBDQ Systemic Symptoms	19.13 (5.36)	26.67 (5.11)	26.73 (3.97)	25.44 (6.63)
IBDQ Emotional Status	61.53 (13.26)	71.27 (6.84)	73.27 (6.94)	70.09 (14.97)
IBDQ Social Functioning	24.33 (6.50)	29.93 (3.65)	30.80 (3.51)	28.78 (7.06)
HDRS	9.47 (5.58)	5.87 (5.85)	5.27 (4.15)	6.94 (7.55)
BDI	8.07 (6.86)	5.00 (5.79)	5.53 (6.31)	3.99 (3.18)
SCL-90 Depression	1.91 (0.71)	1.68 (0.64)	1.55 (0.32)	1.41(0.32)
SCL-90 Anxiety	0.95 (0.30)	0.89 (0.20)	0.80(0.09)	0.83 (0.10)

The results were presented as mean (*sd*).

HBI: Harvey-Bradshaw Index, CDAI: Crohn's Disease Activity Index, MFI: Multidimensional Fatigue Inventory, IBDQ: Inflammatory Bowel Disease Questionnaire, HDRS: Hamilton Depression Rating Scale, BDI: Beck Depression Inventory, SCL-90: Symptom Checklist.

**Table 2:** Immune parameters and TRP/CAA at each time point.

	Baseline	Week 2	Week 4	Week 8
Albumin (mg/dl)	36.06 (6.66)	38.95 (5.54)	39.82 (8.07)	37.53 (7.55)
Albumin %	52.52 (6.91)	55.90 (5.02)	55.61 (5.71)	55.16 (4.93)
α_1_ (mg/dl)	5.72 (0.94)	5.37 (0.99)	5.45 (1.20)	5.16 (1.21)
α_1_%	8.53 (1.86)	7.86 (1.82)	7.85 (2.07)	7.71 (1.68)
α_2_ (mg/dl)	8.86 (1.07)	7.71 (1.08)	8.15 (1.40)	8.04 (1.39)
α_2_%	12.82 (1.55)	11.14 (1.38)	11.48 (1.55)	12.01 (1.58)
β (mg/dl)	7.23 (0.97)	7.23 (1.16)	7.33 (1.29)	6.90 (1.37)
β%	10.73 (1.80)	10.50 (1.90)	10.16 (1.87)	10.29 (1.74)
γ (mg/dl)	10.28 (3.15)	10.30 (2.96)	10.67 (2.99)	10.13 (2.93)
γ%	14.97 (3.88)	14.63 (3.45)	14.81 (3.16)	14.82 (3.40)
Total protein (mg/dl)	68.25 (6.93)	69.68 (7.30)	71.03 (11.91)	67.90 (11.17)
Transferrin (mg/dl)	256.79 (56.63)	271.28 (42.34)	281.29 (49.33)	257.75 (55.58)
TRP/CAA	0.11 (0.02)	0.11 (0.02)	0.12 (0.01)	0.11 (0.02)
Zinc (µg/dl)	95.30 (16.24)	91.96 (16.31)	96.36 (14.14)	89.06 (23.61)

The results were presented as mean (*sd*).

TRP/CAA: tryptophan/competing aminoacids.

**Figure 1:**
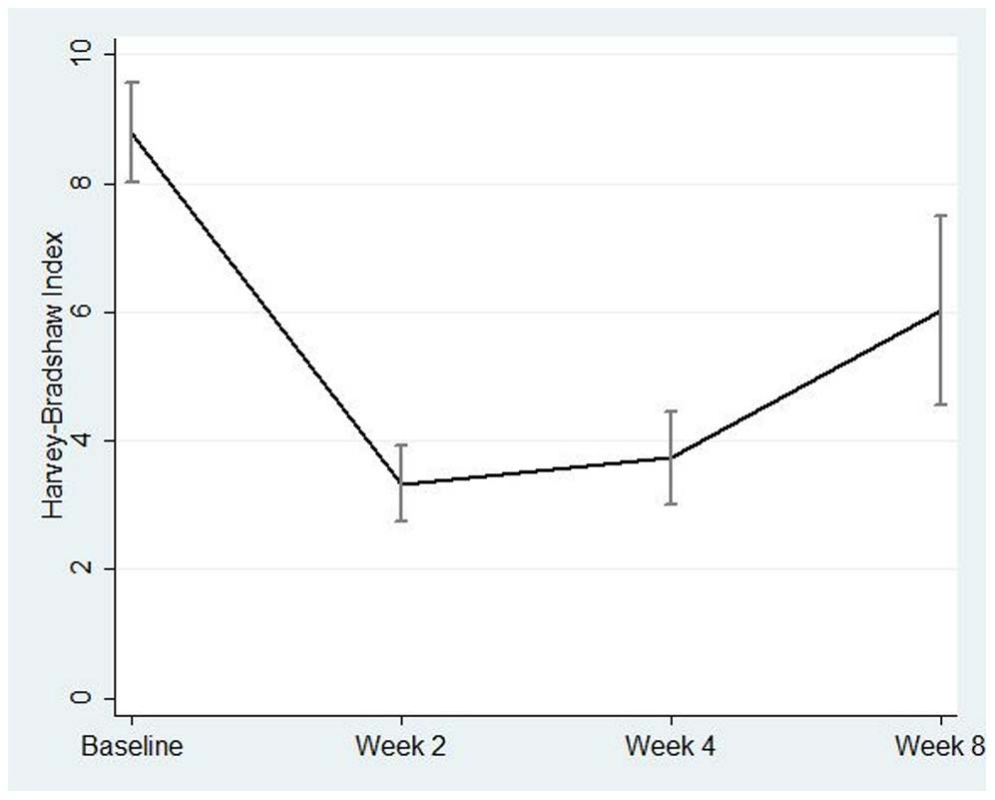
Overtime course of Harvey Bradshaw Index. The line represents the mean score and the error bars represent the standard error at each time point.

CDAI and HBI scores significantly decreased compared to baseline after anti-TNF-α infusion at all time points (Table 3), and the association between these two measures over time was highly significant (B = 18.45, p<0.001). Because of this high correlation, HBI scores were used in further analyses (Figure 1), as this was available at all four time points (CDAI being available only at three time points).

**Table 3:** Effects of anti-TNF-α infusion on disease activity, fatigue, quality of life and mood compared to baseline reference value.

	Week 2		Week 4		Week 8		Time (linear trend)	
	B	P_Simes_	B	P_Simes_	B	P_Simes_	B	P
HBI	−5.57	<0.001	−5.17	<0.001	−2.87	0.006	−0.77	NS
CDAI	NA	NA	−128.8	<0.001	−85.99	<0.001	−33.77	<0.001
MFI physical	−2.33	0.027	−1.67	NS	−2.01	NS	−0.53	NS
MFI mental	−2.20	0.012	−1.13	NS	−0.36	NS	−0.00	NS
IBDQ bowel	16.26	<0.001	16.93	<0.001	11.31	<0.001	3.46	<0.001
IBDQ systemic	7.53	<0.001	7.60	<0.001	6.31	<0.001	1.90	<0.001
IBDQ emotional	9.73	0.001	11.73	<0.001	8.55	0.003	2.77	0.008
IBDQ social	5.60	<0.001	6.47	<0.001	4.45	0.002	1.42	0.008
HDRS	−3.60	0.005	−4.20	0.001	−2.53	NS	−0.82	NS
BDI	−3.07	0.002	−2.53	0.009	−2.81	0.005	−0.81	0.016
SCL-90 Depression	−3.00	0.019	−4.67	<0.001	−5.08	<0.001	−1.71	<0.001
SCL-90 Anxiety	−0.80	NS	−1.40	0.004	−0.93	NS	−0.34	0.032

HBI: Harvey-Bradshaw Index, CDAI: Crohn's Disease Activity Index, MFI: Multidimensional Fatigue Inventory, IBDQ: Inflammatory Bowel Disease Questionnaire, HDRS: Hamilton Depression Rating Scale, BDI: Beck Depression Inventory, SCL-90: Symptom Checklist.

### Effects of anti-TNF-α on fatigue, quality of life and mood

Administration of anti-TNF-α significantly increased scores on all dimensions of quality of life and reduced depression scores measured by the HDRS, BDI and SCL-90 (Table 3). Anxiety, measured by the SCL-90, was significantly decreased only at one time point, namely 4 weeks after infusion. Physical and mental fatigue was decreased only at 2 weeks after infusion. Because of the impact that disease activity may have on mood, analyses were performed again corrected for HBI score (Table 4). IBDQ scores with respect to bowel symptoms, systemic symptoms and social functioning remained significantly increased with respect to baseline. In addition, depressive symptom score measured by SCL-90 remained significantly decreased at 4 and 8 weeks after infusion, and also showed a significant reduction linear trend over time. Reductions in fatigue scores, BDI scores, HDRS scores and SCL-90 anxiety scores no longer were significant after correction for disease activity.

**Table 4:** Effects of anti-TNF-α on fatigue, quality of life and mood compared to baseline scores, corrected for disease activity.

	Week 2		Week 4		Week 8		Time (linear trend)	
	B	P_Simes_	B	P_Simes_	B	P_Simes_	B	P
MFI physical	0.72	NS	1.17	NS	−0.41	NS	−0.17	NS
MFI mental	0.81	NS	1.68	NS	1.35	NS	0.45	NS
IBDQ bowel	9.12	<0.001	10.28	<0.001	7.44	<0.001	1.85	0.006
IBDQ systemic	3.49	0.012	3.84	0.004	4.17	<0.001	1.14	0.003
IBDQ emotional	3.25	NS	5.71	NS	5.19	NS	1.66	NS
IBDQ social	3.07	NS	4.16	0.007	3.40	0.014	0.98	0.024
HDRS	0.25	NS	−0.61	NS	−0.42	NS	−0.22	NS
BDI	−1.58	NS	−1.16	NS	−2.07	NS	−0.53	NS
SCL-90 Depression	−1.97	NS	−3.72	0.016	−4.58	0.001	−1.51	<0.001
SCL-90 Anxiety	−0.10	NS	−0.73	NS	−0.51	NS	−0.21	NS

MFI: Multidimensional Fatigue Inventory, IBDQ: Inflammatory Bowel Disease Questionnaire, HDRS: Hamilton Depression Rating Scale, BDI: Beck Depression Inventory, SCL-90: Symptom Checklist.

### Effects of anti-TNF-α on immune parameters and TRP/CAA

Table 5 shows the changes in immune parameter and TRP/CAA ratio compared to baseline after anti-TNF-α infusion. Serum transferrin, albumin and the percentage of the albumin fraction were significantly increased after infusion, whereas α_1_ and α_2_ fractions and the percentages of α_1_, α_2_ and β fractions were significantly decreased. There was no variation in TRP/CAA ratio compared to baseline after anti-TNF-α infusion. A higher HBI score was associated significantly with a lower TRP/CAA ratio (B = − 0.0009, p = 0.031) over time. There were no significant associations between changes in immune parameters over time and the TRP/CAA ratio (results not shown).

**Table 5:** Effects of anti-TNF-α infusion on immune parameters and TRP/CAA compared to baseline reference value.

	Week 2		Week 4		Week 8		Time (linear trend)	
	B	P_Simes_	B	P_Simes_	B	P_Simes_	B	P
**Albumin**	2.88	NS	3.76	**0.005**	1.47	NS	0.53	NS
**Albumin %**	3.38	**<0.001**	3.10	**<0.001**	2.65	**<0.001**	0.77	**0.001**
**α_1_**	−0.35	NS	−0.27	NS	−0.56	**0.009**	−0.16	**0.019**
**Log α_1_%**	−0.09	**0.005**	−0.09	**0.003**	−0.10	**0.001**	−0.03	**0.002**
**α_2_**	−1.15	**<0.001**	−0.71	**0.004**	−0.82	**0.001**	−0.20	**0.034**
**Log α_2_%**	−0.17	**<0.001**	−0.15	**<0.001**	−0.10	**<0.001**	−0.03	**0.016**
**β**	−0.00	NS	0.09	NS	−0.33	NS	−0.09	NS
**β%**	−0.23	NS	−0.57	**0.003**	−0.44	**0.020**	−0.17	**0.007**
**γ**	0.01	NS	0.39	NS	−0.15	NS	−0.01	NS
**γ%**	−0.34	NS	−0.17	NS	−0.15	NS	−0.03	NS
**Total protein**	1.42	NS	2.78	NS	−0.35	NS	0.03	NS
**Log transferrin**	0.07	NS	0.10	**0.010**	0.01	NS	0.01	NS
**Zinc**	−3.34	NS	2.97	NS	−6.24	NS	−1.28	NS
**TRP/CAA**	0.00	NS	0.01	NS	0.00	NS	0.00	NS

TRP/CAA: tryptophan/competing aminoacids.

### Effects of changes in TRP/CAA ratio and immune parameters over time on depressive symptoms

**Figure 2:**
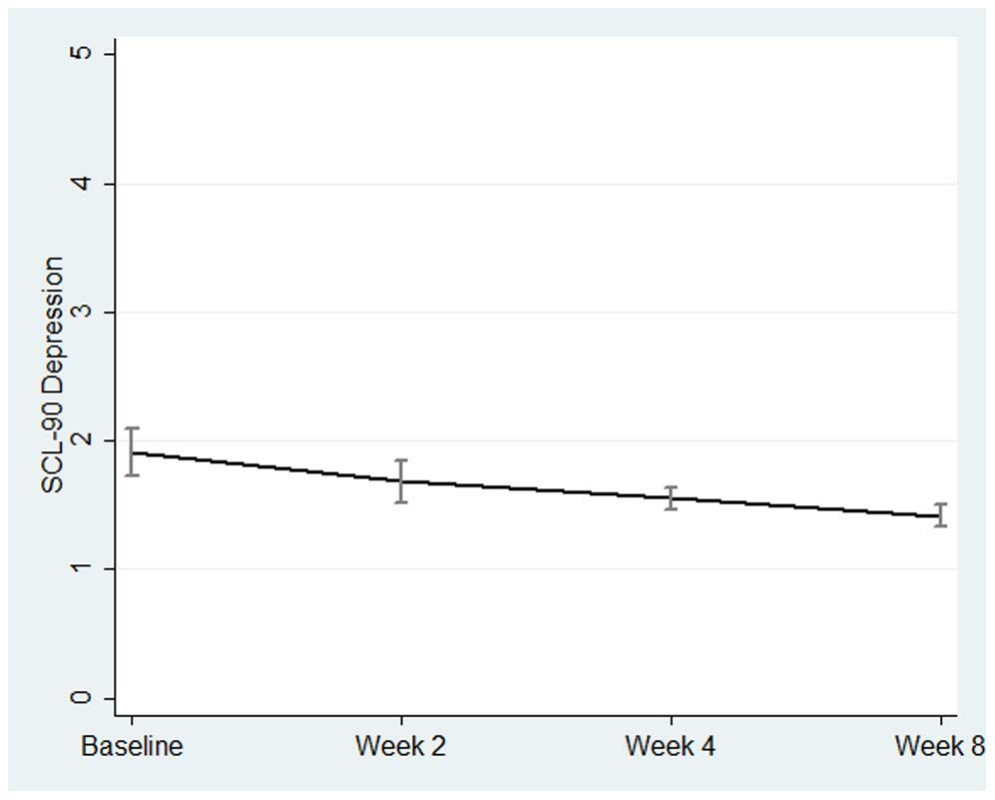
Overtime course of SCl-90 Depression. The line represents the mean score and the error bars represent the standard error at each time point.

As the SCL-90 depression score was most sensitive to mood changes after infusion of anti-TNF-α (Figure 2), the association between mood changes and immune parameters was examined in more detail. Table 6 shows the association over time between SCL-90 depression scores on the one hand and immune parameters as well as TRP/CAA on the other, before and after correction for HBI scores. Overall percentage of γ was associated with SCL-90 depression scores over time (B = 1.15, p_Simes_ = 0.001). After correction for disease activity, both level of γ fraction and percentage of γ were associated with SCL-90 depression scores over time (B = 0.90, p_Simes_ = 0.005 and B = 1.18, p_Simes_ = 0.001).

**Table 6:** Association between SCL-90 depression scores and immune parameters and TRP/CAA over time.

	SCL-90 Depression^a^		SCL-90 Depression^b^	
	B	P_Simes_	B	P_Simes_
**Albumin**	−0.07	NS	0.09	NS
**Albumin %**	−0.46	NS	−0.19	NS
**α_1_**	0.11	NS	0.09	NS
**Log α_1_%**	−4.25	NS	−5.82	NS
**α_2_**	1.26	NS	0.63	NS
**Log α_2_%**	6.98	NS	−2.75	NS
**β**	0.56	NS	0.98	NS
**β%**	−0.34	NS	−0.45	NS
**γ**	0.69	NS	0.90	**0.005**
**γ%**	1.15	**0.001**	1.18	**0.001**
**Total protein**	0.07	NS	0.11	NS
**Log transferrin**	−9.67	NS	−4.35	NS
**Zinc**	0.03	NS	0.04	NS
**TRP/CAA**	17.70	NS	48.21	NS

a without correction for disease activity ^b^corrected for disease activity

SCL-90: Symptom Checklist, TRP/CAA: tryptophan/competing aminoacids.

### Effects of current/past depressive disorder on TRP/CAA ratio and immune parameters over time

After correction for disease activity, overall levels of zinc (B = −24.28, p_Simes_<0.001) albumin fraction (B = −7.02, p_Simes_<0.001) and the percentage of the albumin fraction (B = −7.56, p_Simes_<0.001) were significantly lower and levels of α_2_ (B = 1.15, p_Simes_<0.001), β (B = 0.64, p_Simes_ = 0.005) and γ (B = 2.02, p_Simes_ = 0.028) fractions and those of the percentages of α_2_ (B = 0.18, p_Simes_<0.001), β (B = 1.23, p_Simes_ = 0.002) and γ (B = 3.39, p_Simes_<0.001) fractions were significantly higher in subjects with a current/past depressive disorder. Current/past depressive disorder had no influence on overall TRP/CAA ratio.

## Discussion

To the best of our knowledge, this is the first prospective study investigating the impact of anti-TNF-α (infliximab) infusion on disease activity, quality of life, fatigue and depressive symptoms along with its possible relation to immune parameters and the TRP/CAA ratio in CD patients. The principal findings of this study were as follows: (i) scores of depression scales were decreased after anti-TNF-α infusion and this effect was to a degree, but not entirely, reducible to disease activity; (ii) there was no change in the TRP/CAA ratio after anti-TNF-α infusion and neither scores of depression scales, nor immune parameters were associated with TRP/CAA ratio; (iii) immune activation was higher in patients with current/past depressive disorder.

The association between the immune system and depression comes from several lines of evidence, including the induction of sickness behavior (which resembles core features of major depression in patients such as sleep disturbances, anergia and anhedonia) in animals treated with inflammatory agents, and the high comorbidity of inflammation related medical disorders - e.g. CD, rheumatoid arthritis, psoriasis and treatments with immune modulators-with psychopathology [13], [14], [41] Moreover, it was shown that treatment of CD with anti-TNF-α reduces not only disease activity but also depressive symptoms [8], [29], [42]. The present findings, showing decrement in depressive symptom scores measured by SCL-90, BDI, and HDRS in patients with CD after anti-TNF-α treatment, are in agreement with these previous reports. Considering that depressive symptom scores measured by SCL-90 remained significantly decreased after correction for HBI scores compared to baseline, it is plausible that the decrease in depressive symptoms maybe mediated in part through changes in the immune system rather than only through reductions in psychological distress due to reduced disease activity. The differences between these three scales are likely related to differences in the constructs underlying the scales. [43] The HDRS in fact taps into various symptom groups that may be associated with the nuclear depression syndrome, particularly somatic symptoms. Although the HDRS is widely used, the SCL-90 is a more unidimensional measure of the nuclear depression syndrome, whereas the BDI assesses attitudes and cognitions which are mostly stable overtime. Given these differences, the SCL-90 was chosen for further analysis.

In addition, it was found that decrements in depressive symptom scores after anti-TNF-α infusion followed the reduction of inflammation as defined by an increase in negative APPs (albumin) and a decrease in positive APPs (α_1_ and α_2_ fractions). Research suggests that an imbalance of the immune system plays a role in depression through several interacting mechanisms [13]. There is some evidence that serotonin neurotransmission may play a role in the pathogenesis of depression and given the fact that TRP depletion decreases mood in vulnerable people, much attention has been focused on the degradation of TRP by the indoleamine 2,3 dioxygenase (IDO) enzyme, which is predominantly induced by IFN-γ and TNF-α [13], [44]. In contrast to earlier studies showing decreased TRP/CAA ratios in patients with depression [21], [24], [25], [26], [27] and correlations between depressive symptoms and TRP levels in IFN-γ induced depression [45], [46], we did not find any increase in TRP/CAA ratio over time and there was no association over time between SCL-90 depressive symptom scores and TRP/CAA ratio after infusion of anti-TNF-α. Considering the earlier findings showing decreased TRP/CAA ratio in IFN-γ induced depression,the fall in TRP may explain the role of immune mediators in depression,albeit partially. It has been argued that IFN-γ induced depression is associated with induction of IDO that increases levels of kynurenine (KYN), which in turn leads to formation of neurotoxic metabolites, 3-hydroxykynurenine and quinolinic acid, rather than just TRP degradation by itself [13], [39], [44], [47], [48], [49]. Furthermore, it has been demonstrated there was no relationship between response to antidepressant treatment and TRP/CAA ratio in a large group of patients with major depression [50]. In the light of these findings, it can be speculated that the reduction in depressive symptom scores in our sample is maybe associated with restoration of balance in KYN pathway rather than changes in TRP/CAA ratio.

Similar to the findings of the current study demonstrating invariant TRP/CAA ratio after treatment with anti-TNF-α, it was shown adalimumab, which is also a TNF-α antagonist, exerts its immune-suppressant effect without influencing IDO activity and TRP levels in patients with rheumatoid arthritis [51]. Furthermore, the present study also failed to find an association between immune parameters and TRP/CAA ratio over time. Consequently, it may be reasoned that TNF-α antagonists exert effects on disease activity and depressive symptoms through several different pathways, for example nuclear factor kappa B modulation of cell survival and apoptosis, corticotrophin releasing factor, vasopressin, brain-derived neurotrophic factor [13], [14], [52], [53].

In the current study it was demonstrated that, even after correction for disease activity, levels and percentages of positive APPs (α_2_, β, γ) were higher, while levels and percentages of albumin and levels of zinc were lower in patients with a current or past depressive episode. In other words, an inflammatory reaction was more evident in patients with depression, and not solely dependent on disease activity. The association between SCL-90 depression scores and γ fraction after correction for disease activity also fortifies this notion. There are numerous studies showing that depression exacerbates CD and predicts lower remission rates in CD [5], [6], [7], [12]. In order to clarify the additive effect of depression on immune activation and disease activity in CD, studies comparing immune parameters between CD patients with and without depression are required.

In agreement with previous studies evaluating CD patients, improvement in quality of life after anti-TNF-α infusion was demonstrated by increases across all domains of IBDQ [8], [54], [55]. Likewise, in line with previous studies, anti-TNF-α infusion decreased physical and mental fatigue at week 2. Nevertheless, decreases in fatigue scores were no longer significant after correction for disease activity. Contrary to this latter finding, it has been suggested that fatigue in CD is secondary to depression rather than a primary manifestation of disease activity [56].

The strength of this prospective study is that it has not only analysed the effect of anti-TNF-α treatment on depressive symptoms in CD patients, but also concentrated on the underlying immune mechanism by measuring TRP/CAA ratio and immune parameters with corrections for disease activity. Although the study design allowed us to use patients as their own controls over a time period of 8 weeks, the sample size was rather small which may have caused type-II errors. We carefully evaluated the presence of axis I mental disorder using the Structured Clinical Interview for DSM-IV axis I Disorders Version 5.0 in this sample. Two patients fulfilled criteria for lifetime depressive disorder and two for current depressive disorder with melancholic features. Since the presence of depressive disorder is a likely confounder, all analyses were corrected for this variable. We did not specifically collect information about patients' psychotropic treatment plan, however this likely overlaps with presence of mental disorder. Other limitations also apply. In the present study, TRP availability to the brain was estimated by the peripheral TRP/CAA ratio. Various previous studies measured brain tryptophan availability using the same TRP/CAA ratio. [21], [24], [25], [26], [28], [39], [50], [57]. Given that plasma TRP concentrations correlate poorly with those in cerebrospinal fluid (CSF), the TRP/CAA ratio gives us a more reliable measure of brain TRP availability taking into account the fact that CAA is competing for the cerebral uptake of TRP [57]. For a more direct approach, TRP and its metabolites would have to be measured in CSF, which might provide a better estimate [58]. However, lumbar puncture in these patients ethically would be considered invasive, given lifetime chronic illness requiring major medical diagnostic testing and interventions over the course of the illness. As explained previously, measurement of IDO activity, along with its products and the ratio between neurotoxic and neuroprotective metabolites may allow for a more direct test of the question whether a decrease in depressive symptom scores after anti-TNF-α is due to changes in TRP and its metabolites, or whether several other mechanisms are involved. Another approach to understand the effect of TNF-α antagonists on depression may be to evaluate the effect of anti-TNF-α treatment on treatment response and immune markers in patients with depression. This approach may help to eliminate other potential confounders originating from the nature of autoimmune disorders like CD requiring use of concomitant medication influencing immune parameters. Ongoing studies evaluating the efficacy of drugs with antagonist properties at TNF-α, namely infliximab and minocycline, for treatment of treatment resistant depression and bipolar depression along with the relationship between efficacy and inflammatory markers will hopefully shed more light on the association between TNF-α system and depression [59], [60].

Notwithstanding its limitations, this study does suggest that anti-TNF-α infusion in patients with CD reduces depressive symptoms, in part independently of disease activity, and that the effect on depressive symptoms is not associated with immune-induced changes in TRP availability to the brain, as estimated indirectly by serum TRP/CAA ratio.
